# Unresolved Grief Resurfacing: managing delayed perinatal grief after subsequent birth

**DOI:** 10.1192/j.eurpsy.2025.2388

**Published:** 2025-08-26

**Authors:** B. Arribas-Simon, O. Martin-Santiago, C. Alario-Ruiz, O. Segurado-Martin, M. J. Mateos-Sexmero, P. Pando-Fernandez, P. Martinez-Gimeno, B. Rodriguez-Rodriguez, N. Navarro-Barriga, P. Andres-Olivera, M. Calvo-Valcarcel, M. Andreo-Vidal

**Affiliations:** 1 Psychiatry, Hospital Clinico Universitario, Valladolid; 2 Psychiatry, CAUSA, Salamanca, Spain

## Abstract

**Introduction:**

Delayed perinatal grief occurs when the grieving process for a lost baby is reactivated after the birth of a healthy child. This case presents a 39-year-old mother who, after losing her first baby at 36 weeks due to Patau syndrome, experienced delayed grief following the birth of a full-term baby two years later. Despite receiving one psychological consultation at the time of the loss, the lack of follow-up contributed to the reactivation of her grief postpartum, presenting with sadness and anxiety.

**Objectives:**

- To describe the process of delayed perinatal grief in a mother who lost a baby due to Patau syndrome.
- To evaluate the psychological impact of the lack of follow-up after the loss on the subsequent development of reactivated grief.
- To propose therapeutic interventions for the management of mothers experiencing delayed perinatal grief.

**Methods:**

We present the case of a 39-year-old mother who lost a baby at 36 weeks of gestation due to Patau syndrome. Following the loss, she received a single psychological consultation with no further follow-up. Two years later, she gave birth to a healthy baby at 40 weeks, and six weeks after delivery, she was referred to psychiatry due to symptoms of profound sadness and anxiety, consistent with delayed perinatal grief. The patient was evaluated by the psychiatry team and began treatment with psychological intervention and pharmacological management when necessary.

**Results:**

The psychiatric intervention led to a gradual improvement in symptoms of sadness and anxiety. The patient responded favorably to psychological treatment, incorporating cognitive-behavioral therapy techniques to manage grief. However, feelings of sadness persisted on dates related to the previous loss. Ongoing emotional support was crucial for the recovery process.

**Conclusions:**

Delayed perinatal grief can reactivate after the birth of a new child, especially in cases where the original loss was not adequately followed up. Proper psychological support is essential to help mothers process their grief and prevent long-term emotional complications.

**Disclosure of Interest:**

None Declared

